# A Silicon Photonic Data Link with a Monolithic Erbium-Doped Laser

**DOI:** 10.1038/s41598-020-57928-5

**Published:** 2020-01-24

**Authors:** Nanxi Li, Ming Xin, Zhan Su, Emir Salih Magden, Neetesh Singh, Jelena Notaros, Erman Timurdogan, Purnawirman Purnawirman, Jonathan D. B. Bradley, Michael R. Watts

**Affiliations:** 1 Research Laboratory of Electronics, Massachusetts Institute of Technology, 77 Massachusetts Avenue, Cambridge, MA 02139 USA; 2 John A. Paulson School of Engineering and Applied Science, Harvard University, 29 Oxford Street, Cambridge, MA 02138 USA; 3 Present Address: Institute of Microelectronics, Agency for Science, Technology and Research (A*STAR), 2 Fusionopolis Way, Singapore, 138634 Singapore; 4 Present Address: Analog Photonics, 1 Marina Park Drive, Boston, MA 02210 USA; 5 Present Address: Department of Electrical and Electronics Engineering, Koç University, Sarıyer, İstanbul 34450 Turkey; 6 Present Address: Department of Engineering Physics, McMaster University, 1280 Main Street West, Hamilton, Ontario L8S 4L7 Canada

**Keywords:** Electrical and electronic engineering, Integrated optics

## Abstract

To meet the increasing demand for data communication bandwidth and overcome the limits of electrical interconnects, silicon photonic technology has been extensively studied, with various photonics devices and optical links being demonstrated. All of the optical data links previously demonstrated have used either heterogeneously integrated lasers or external laser sources. This work presents the first silicon photonic data link using a monolithic rare-earth-ion-doped laser, a silicon microdisk modulator, and a germanium photodetector integrated on a single chip. The fabrication is CMOS compatible, demonstrating data transmission as a proof-of-concept at kHz speed level, and potential data rate of more than 1 Gbps. This work provides a solution for the monolithic integration of laser sources on the silicon photonic platform, which is fully compatible with the CMOS fabrication line, and has potential applications such as free-space communication and integrated LIDAR.

## Introduction

With the increasing demand for data communication bandwidth, silicon photonic technology has been extensively studied in integrated optical circuits to overcome the limits of electrical interconnects. Silicon is the material of choice for both microelectronic circuits and integrated photonic components. The high refractive index contrast between silicon and the silicon dioxide cladding enables highly compact photonic devices. Additionally, the compatibility with the mature complementary-metal-oxide-semiconductor (CMOS) fabrication technology allows for low-cost and high-volume production of silicon photonic devices, including large-scale phased arrays^1–3^, low power modulators^4,5^, ultra-wideband multiplexers^6^, and waveguides for supercontinuum generation^7,8^. The demonstration of these devices makes silicon photonic an ideal platform for system-on-chip optical interconnects.

Yet, all of the optical data links demonstrated so far used either heterogeneously integrated lasers^9–11^ or external laser sources^12–15^. One of the main challenges in a true monolithic data link is the indirect bandgap of silicon, rendering it unsuitable as a lasing medium. To overcome this problem, different approaches have been used to realize laser sources on silicon, including heterogeneous integration or direct growth of III-V semiconductors^16–18^, stimulated Raman emission in silicon^19,20^, germanium (Ge) and germanium-tin (GeSn)^21–23^, and rare-earth-doped thin films^24–28^. Among these approaches, deposition of rare-earth-doped aluminum-oxide (Al_2_O_3_:RE^3+^) glass as the gain medium^29^ has proven to be effective for advanced circuits on silicon, due to several key advantages. First, the rare-earth-doped glass can be deposited on silicon wafers as a single-step back-end-of-line process^30^, which enables monolithic, scalable integration and potential for low-cost high-volume mass production. Second, rare-earth-ion-based lasers can provide narrow intrinsic linewidths since the optical pumping process introduces no free carriers^25,31–33^. Furthermore, the low thermo-optic coefficient of the host medium (Al_2_O_3_) enables laser operation over a wide temperature range^34,35^, important for control-free WDM systems^36^. Both erbium- and thulium-doped lasers are able to achieve high optical output power^37,38^, which is able to meet the challenging power budget in LIDAR systems^39,40^ or ultrafast pulsed lasers^41^. Finally, common rare-earth materials, such as erbium, thulium, and holmium, have wide emission spectra enabling large wavelength range coverage^32,42–44^ and the potential for short pulse generation through mode-locking^26,45–49^. In addition, the effectiveness of rare-earth-doped lasers on silicon is further enhanced with the availability of compact un-cooled pump laser diodes^50^ and recent advances in photonics packaging^51^, which makes the co-package of the optical pump lasers easy to implement and achieve system-level compactness. Hence, besides the advantage of providing narrow laser linewidth, the advances in pump diode packaging offer compact solution for the optical pump to power several rare-earth-doped lasers simultaneously.

Using CMOS-compatible fabrication methods, rare-earth-ion-based monolithically integrated lasers have been demonstrated across near-infrared wavelengths at 1.0, 1.5, 1.8, 2.1 µm using ytterbium^52,53^, erbium^25,27,54^, thulium^37,55^, and holmium^56^ doped Al_2_O_3_ glass as gain media, respectively. These lasers use silicon-nitride (Si_3_N_4_) cavities, as Si_3_N_4_ has high transparency and low loss from near-IR to the mid-IR wavelength regime^57,58^. This approach provides a mature wafer-scale waveguide platform for both passive and nonlinear silicon photonic devices^59–61^. Nevertheless, the integration of rare-earth-ion-based lasers on a full silicon photonic platform has proven to be challenging due to the added design and fabrication complexity, and the fact that the high Al_2_O_3_:RE^3+^ deposition temperature tends to damage the active devices within the platform^30^. Up until now, rare-earth-doped lasers on an active silicon-on-insulator (SOI) wafer platform, which enables integration of lasers with active silicon photonic circuits, have only been recently demonstrated with an optical phased array^39^. Such system demonstrated earlier has limited integration complexity without signal modulator and photodetector as receiver.

In this work, an optical data link using a monolithically integrated rare-earth-doped laser as a light source on an SOI wafer is demonstrated for the first time. An erbium-doped distributed Bragg reflector (DBR) laser, a silicon microdisk modulator and a Ge detector are monolithically integrated and used for signal generation, modulation and detection, respectively. By using a backend step for Al_2_O_3_:Er^3+^ deposition at an on-chip temperature of 310 °C, we enabled CMOS-compatible fabrication of all the active and passive silicon photonic components on a single chip. The system demonstrates data transmission as a proof-of-concept at kHz speed level, and the potential data rate of more than 1 Gbps.

## Results

### Integrated system design

**Figure 1:**
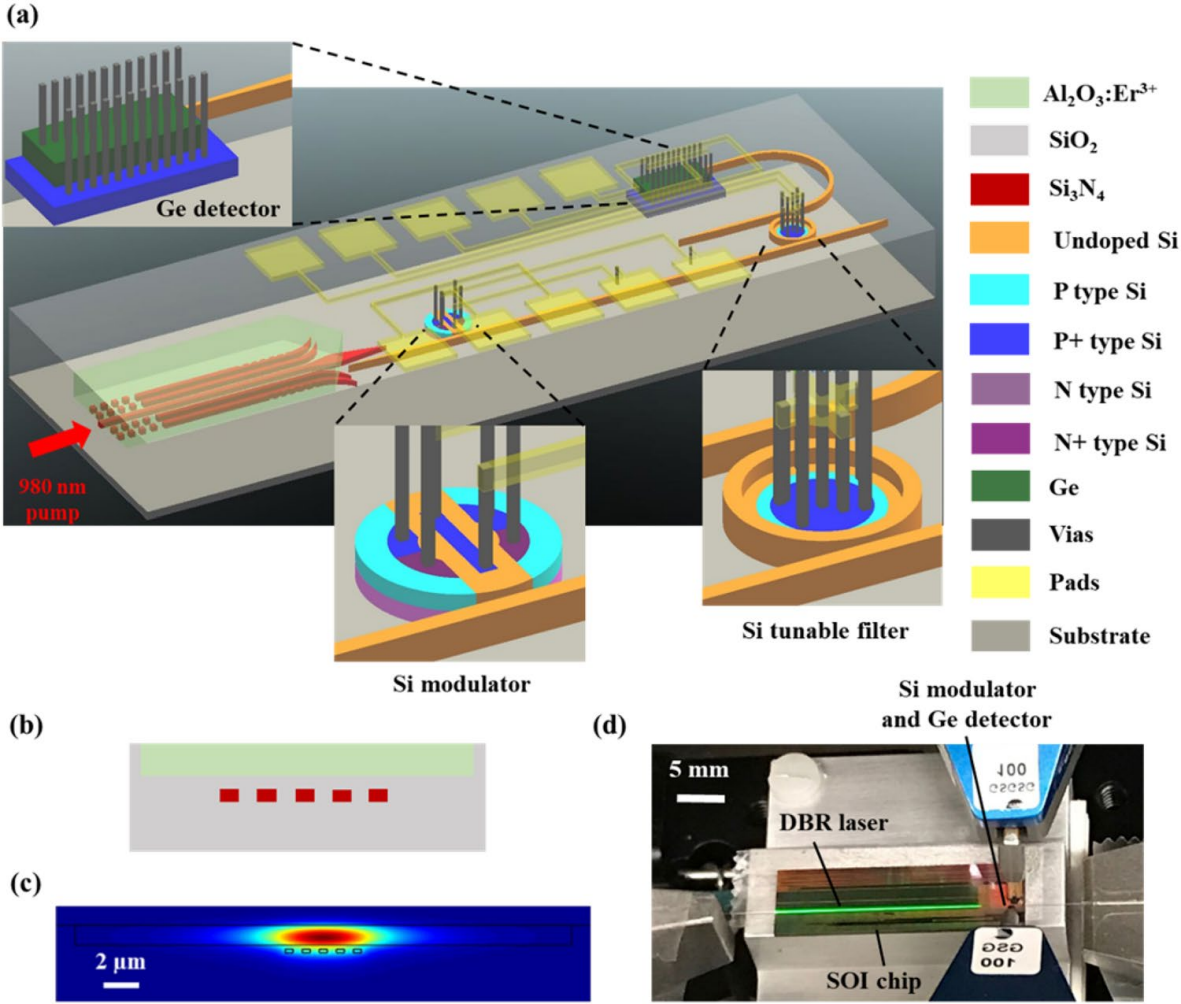
(**a**) Schematic of the photonics link, including the integrated erbium-doped laser, silicon microdisk modulator, silicon microring tunable filter, and germanium (Ge) photodetector (drawing not to scale). (**b**) Integrated erbium-doped distributed Bragg reflector (DBR) laser gain waveguide cross section. (**c**) The electric field intensity of the fundamental mode for the DBR laser gain waveguide. (**d**) The fabricated system on the test setup, showing green color fluorescence from the Al_2_O_3_:Er^3+^ laser waveguide excited by the 980 nm pump.

A schematic diagram of the system is shown in Fig. 1(a). It mainly consists of four components: an erbium-doped DBR laser source, a silicon microdisk modulator, a silicon tunable filter, and a Ge photodetector.

The waveguide cross section of the DBR laser is shown in Fig. 1(b). The width and gap of the Si_3_N_4_ pieces are selected to be 450 nm and 400 nm, respectively, to provide large mode confinement factors for both the 980 nm pump and 1560 nm laser signal modes within the Al_2_O_3_:Er^3+^ film. Figure 1(c) shows the fundamental transverse electric (TE) mode intensity profile of the 1560 nm signal. The height of each Si_3_N_4_ piece is 200 nm. The gap between the Si_3_N_4_ and Al_2_O_3_:Er^3+^ layer is 200 nm. A 1100-nm thick Al_2_O_3_:Er^3+^ film is deposited on top of the chip to provide gain. The DBR cavity is formed by Si_3_N_4_ grating pieces on both sides of the laser gain waveguide, with a duty cycle of 0.5 and a period of 493 nm. At the pump input, 4 side Si_3_N_4_ pieces in the gain waveguide act as gratings to provide optical feedback at lasing wavelength, as shown in Fig. 1(a). At the signal output, the widths of both leftmost and rightmost Si_3_N_4_ piece in the gain waveguide are reduced from 450 nm to 350 nm. The periodic variation of the original and the reduced Si_3_N_4_ width forms grating and creates feedback for lasing signal. The schematic is also illustrated in Fig. 1(a). The coupling coefficient (κ) is calculated to be 4.5 × 10^3^ m^−1^ and 5.7 × 10^2^ m^-1^ at the pump input side and lasing signal output side respectively, in order to provide sufficient feedback for lasing and reasonable signal power for output. The total length of the DBR laser is 2 cm, limited by the maximum length of the chip. At the end of the DBR laser, a transition is designed to adiabatically couple the mode from the DBR gain waveguide into the mode of a waveguide in the Si_3_N_4_ layer, with a waveguide width of 1.5 μm. At the end of Si_3_N_4_ waveguide, the mode is again adiabatically coupled into a waveguide in the silicon layer, with a silicon width of 0.4 μm.

Once the laser mode is adiabatically coupled into the silicon waveguide, it is then evanescently coupled into a silicon microdisk modulator, as shown in the enlarged area of Fig. 1(a). The modulator has a vertical p-n junction with implants of different doping energies (similar to^62,63^). The modulator design is based on the existing component reported earlier with minor modifications^64^, where the extinction ratio and the insertion loss of the silicon microdisk modulator have been reported to be 5 dB and 1 dB, respectively. The vertical junction structure maximizes the overlap of the depletion region with the optical mode, minimizing the power consumption and drive voltage. The modulator is contacted in the center of the disk using n+ and p+ implants and metal vias. Compared with the ridge waveguide structure^65^, the contact of the vertical junction can be made from the interior of the resonator and hence enables a hard outer wall, which minimizes the radiation loss due to the tight bends in a small diameter resonator. The structure further reduces the capacitance and the drive power of the modulator. The microdisk has a radius of 3 μm, which gives a 38 nm free spectral range (FSR). Its electro-optic phase efficiency V_π_**·**L is measured to be 0.61 V**·**cm. The microdisk modulator also has an integrated heater with interior contacts that enables a thermal tuning range of 6 nm and efficiency of 0.86 nm/mW, in order to match the modulator’s resonance with the lasing signal. A similar vertical junction microdisk modulator design reported earlier by our group has a measured electro-optic 3 dB bandwidth of 21 GHz^5^.

After signal modulation, the optical mode is coupled back into the silicon waveguide, and transmitted to the receiver side. A silicon microring filter (similar to^32,66^) is used to filter and couple the modulated signal into the Ge detector. The filter acts as an essential component for reconfiguration of future optical communication network on chip. The zoomed-in views of both the silicon microring tunable filter and the Ge detector are shown in Fig. 1(a). For the tunable filter, an interior-ridge silicon resonator is selected, which introduces a relatively thicker outer wall. The high index contrast at the outer wall enables tight bend of the waveguide without introducing large radiation loss. For tunability of this resonator, an embedded silicon heater is formed within the ridge-etched region, using low- and high-dose p-type implants. The attachment of a silicon heater to the waveguide core directly heats the silicon in a thermally isolated environment (i.e. buried SiO_2_), achieving an efficient thermal tuning of 1 nm/mW. The outer radius of the silicon microring is 3 μm, with an FSR of 35 nm. The position of the doped regions is optimized for minimum absorption due to the embedded heater. The heater resistance is reduced by forming the heater with multiple resistors that are connected in parallel to minimize the drive voltage. The resonance of the tunable filter is thermally tuned to match the erbium-doped DBR laser signal wavelength, select the modulated signal, and couple into the Ge detector.

The coupling from the silicon bus waveguide to Ge detector is achieved by an evanescent taper. This coupling method uses mode evolution to transfer power into the Ge-on-Si region gradually and efficiently with longer taper lengths enabling higher transmission^67^. The Ge is hetero-epitaxially grown into deep oxide trenches on top of a body silicon layer, which is heavily p-doped. The body silicon layer is extended outside the Ge to be contacted. The top of the Ge is implemented by a shallow n-type dopant and contacted directly, forming a vertical p-i-n junction between the top of the Ge layer and the body silicon base layer. The Ge detector has a length of 12 μm and a width of 4 μm. The length is chosen to balance the trade-off between the coupler length and bandwidth. The design is also based on the existing component reported earlier with minor modifications^64,67^, where the 3 dB bandwidth is limited to 40 GHz and responsivity can reach 1 A/W at the designed working wavelength. The full fabricated integrated system excited by a 980-nm laser diode pump is shown in Fig. 1(d). Green fluorescence from the erbium-doped DBR laser waveguide can be observed. Optical pumping used here can provide better linewidth without complex locking loops^31,33^.

### System characterization and discussion

**Figure 2:**
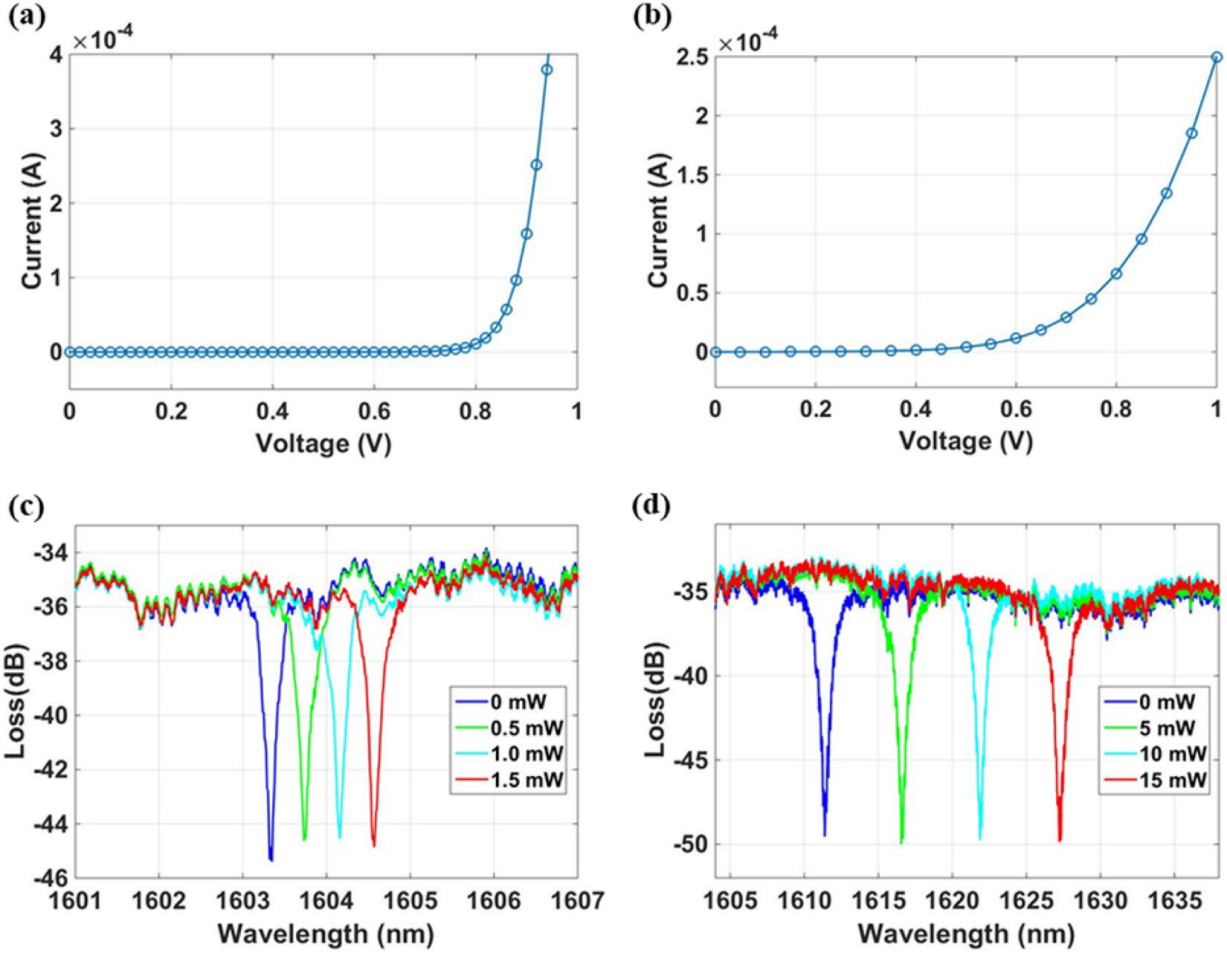
Current-voltage characteristics of the (**a**) vertical junction microdisk modulator and (**b**) Ge photodetector, showing the diode responses. Thermal tuning of the (**c**) microdisk modulator and (**d**) microring filter. Both diode responses and thermal tuning indicate working condition of the system after erbium-doped Al_2_O_3_ thin film deposition at 310 °C.

It is known that post processing of the CMOS wafer beyond 400 °C can compromise the integrity of the metal contacts and vias. In order to find out the proper Al_2_O_3_:Er^3+^ thin film deposition temperature for our fabricated device, successive deposition iterations from high to low temperature were conducted, until the current-voltage (I-V) characteristics of the modulator and Ge photodetector still show the diode response after the deposition. Such temperature was found to be 310 °C on the substrate of the device. More details about the substrate temperature measurement are provided in Supporting Information section 1. To further prove the CMOS compatibility of the back-end-of-line Al_2_O_3_:Er^3+^ thin film deposition, the I-V characteristics of the silicon microdisk modulator and the Ge detector after the deposition are compared with the ones measured from the chip with the same device design and fabrication process before the gain film deposition, showing insignificant degradation. The diode responses of the microdisk modulator and the Ge photodetector after the deposition are presented in Fig. 2(a,b), respectively. Both demonstrate the expected diode response after the deposition and confirm that the metal layer is not damaged by the thermal condition during the deposition. In addition, the thermal tuning of the microdisk modulator and microring filter for detector are tested, as shown in Fig. 2(c,d), respectively. The thermal tuning efficiency of the microdisk modulator and microring filter are measured to be 0.86 nm/mW and 1.0 nm/mW, respectively. Both I-V characteristics and the thermal tuning demonstrated in Fig. 2 indicate that the system is still in working condition after the Al_2_O_3_:Er^3+^ thin film deposition at 310 °C.

Next, an external tunable laser source is used to obtain the passive response of the system. The tunable laser source is used to sweep the wavelength of the input signal. A polarization controller is placed after the external tunable laser source to ensure the input laser signal is coupled into the fundamental TE mode of the DBR laser and silicon waveguide. An optical power meter is used to record the signals at the through port of the detector microring filter. A thermoelectric cooler (TEC) is placed at the bottom of the chip to monitor and stabilize the operating temperature of the system with a feedback loop. A cleaved single-mode HI1060 fiber is used on the input side of the chip to butt-couple the tunable laser signal onto the chip, and a lensed fiber with 3-μm spot size is used to couple out the output signal from the silicon taper at the other end of the chip. A drawing of the measurement setup and more details are provided in Supporting Information section 3.

**Figure 3:**
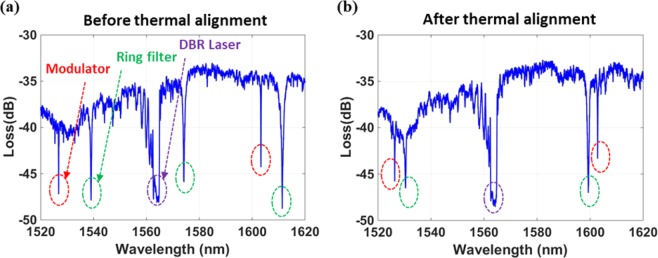
(**a**) Passive response of the link system showing the resonances of the microdisk modulator, detector ring filter, and DBR laser marked in red, green, and purple dotted lines, respectively. (**b**) Passive response of the link after thermal alignment of the resonances of the microdisk modulator, detector ring filter, and DBR laser.

In Fig. 3(a), the resonances marked with red, green, and purple circles are the modulator, detector ring filter, and DBR laser cavity resonances, respectively. The microdisk modulator has a higher total Q-factor than the ring filter in design, therefore, exhibiting a narrower resonance than the microring filter connected to the photodetector. One of the modulator resonances overlaps with the DBR laser cavity resonance, but does not exactly match the laser wavelength. Thermal tuning is then used to match the DBR laser wavelength, the microdisk modulator resonance, and the detector ring filter resonance at 1564 nm, as shown in Fig. 3(b).

**Figure 4:**
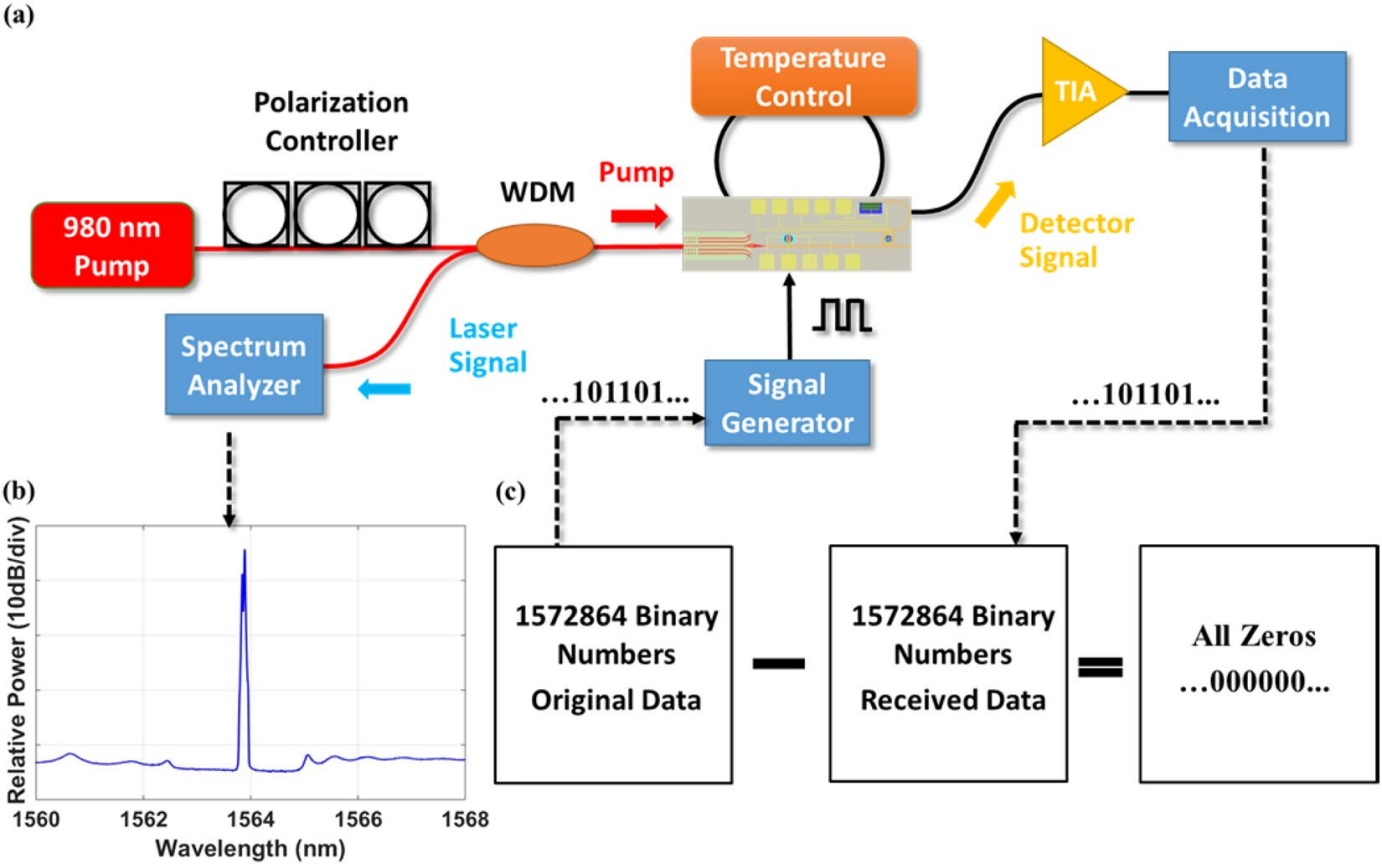
(**a**) Active characterization setup including a 980 nm laser pump source together with a polarization controller to ensure the fundamental TE mode is coupled into the Al_2_O_3_:Er^3+^ DBR laser, an OSA to monitor the DBR laser output, a temperature control feedback loop to modify and monitor the temperature of the system, and a TIA to amplify the electrical signal that is then monitored by the oscilloscope. (**b**) Optical spectrum of the DBR laser recorded by the OSA, showing >30 dB SMSR. (**c**) The subtraction between the original binary data for signal generator and the received binary data from Ge photodetector gives all zero, showing data transmission without error bit.

Following passive response measurement and resonance alignment, the active characterization of the system is conducted using the setup shown in Fig. 4(a). A 980 nm pump source together with a polarization controller is used to couple the pump signal into the fundamental TE mode of the laser gain waveguide. An external WDM is used to filter out the lasing signal from the pump side of the DBR laser and an optical spectrum analyzer (OSA) is used to monitor the signal from the DBR laser. The lasing spectrum is shown in Fig. 4(b), with more than 30 dB side mode suppression ratio (SMSR). The lasing spectrum is recorded by the OSA with a wavelength interval of 0.004 nm. A cleaved HI1060 fiber is used on the input side of the chip to butt-couple the pump onto the chip, with launched pump power of 60 mW. The laser slope efficiency is estimated to be 0.02%, which is limited by the reduced gain film depositon temperature and the roughness at the bottom of the gain waveguide. More details about the laser efficiency improvements and system power budget are provided in Supporting Information section 4. The TEC is used to monitor the operating temperature of the system and stabilize the device temperature by reducing the thermal shift due to the pump power. The electrical modulation signal is applied on the silicon modulator through a high-speed probe. The modulated lasing signal then propagates through the silicon waveguide and couples into the Ge photodetector through the microring filter. Another high-speed probe in contact with the integrated Ge photodetector is used to collect the electrical signal. The photocurrent is sub-micro ampere. In order to record the electrical signal from Ge detector, the collected photocurrent is amplified by an off-chip trans-impedance amplifier (TIA) (SR570 low noise current preamplifier) and then read by a data acquisition card.

Using the integrated data-link system, we demonstrate data transmission. The 1572864 binary numbers to be transmitted are decoded from a color image. To avoid potential thermal drift on the silicon modulator from consecutive “0” or consecutive “1” modulation voltages, each decoded binary number “0” and “1” are further replaced by “01” and “10”, respectively. The generated 3145728 binary numbers are then converted into voltage signals (-0.8 V for “0”, and 1 V for “1”) and applied to the silicon modulator to modulate the laser signal. At the Ge photodetector, the received modulated signal is first collected by a National Instrument (NI) data acquisition card (USB 6361) and then each two adjacent voltage signals are grouped into one pair. By comparing the relative voltage values of two signals, each pair can be easily converted into a binary number (“0” for “01” pattern and “1” for “10” pattern). Finally, the original 1572864 binary numbers are subtracted by the received binary numbers, which gives 1572864 zero numbers, demonstrating data transmission without error bit. The data transmission experiment is conducted at kHz level speed as a proof-of-concept for the first monolithic silicon photonic data link. The speed is mainly limited by the 1 MHz maximum bandwidth of the TIA. Based on the previous experimental results of the silicon modulator and photodetector^64,67^, if the state-of-the-art TIA with GHz bandwidth can be used here or the lasing power can be increased so as to reduce the TIA gain requirement, the signal transmission with higher modulation frequency at GHz level can be readily demonstrated.

**Figure 5:**
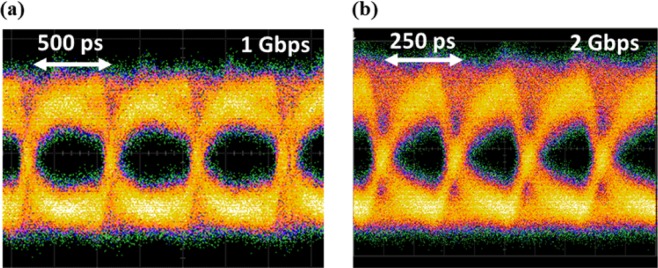
The eye pattern of the data link system (measured using an external laser source with the integrated laser bypassed) under (**a**) 1 Gbps and (**b**) 2 Gbps data stream.

To analyze the potential high-speed capabilities of the system, we bypass the integrated laser and instead use an external high power laser source to inject power from the input side of the link. For this experiment, instead of using an Al_2_O_3_:Er^3+^ gain film, an undoped Al_2_O_3_ thin film is deposited on the chip with the same design to form the passive laser waveguide without signal absorption from the erbium ions. The experimental setup is provided in Fig. S4 of the Supporting Information. A laser source cascaded with a high-power erbium-doped fiber amplifier (EDFA) is used as an external source to provide the laser signal at the wavelength matching with the resonances of the silicon microdisk as well as the microring filter. The amplified laser signal is butt-coupled onto the chip through a cleaved HI1060 fiber, with an estimated on-chip power of 136 mW, which we have demonstrated using similar rare-earth-doped waveguide laser structures^37^. Such power is required, based on the system loss budget provided in Supporting Information section 4, for the Ge detector to generate enough photocurrent for later analysis without using a TIA. A pattern generator is used to provide 1 or 2 Gbps level pseudorandom binary sequence (PRBS) signal to the silicon modulator. A sampling scope is connected to the Ge detector through a bias tee to capture the eye diagram, as shown in Fig. 5(a,b). We observe open eye patterns at 1 and 2 Gbps. As the frequency of the PRBS is increased beyond 3 Gbps, the “eye” becomes unclear, which might be contributed by the loss from the mode transitions and the microring structures. The future work to improve the power budget within the system includes the optimization of the wafer-level laser trench fabrication process, the design for the waveguide transitions, the gain film deposition and the laser cavity design. More details about the high-speed characterization setup and the system loss budget are provided in Supporting Information section 3 and 4 respectively. The speed of this proof-of-concept system can be improved by reducing the system loss and increasing the power coupled into the photodetector.

## Conclusions

In conclusion, we have demonstrated a single-chip optical data link on an SOI wafer fabricated in a standard CMOS foundry. An erbium-doped DBR laser is monolithically integrated as the light source. A reverse-biased vertical junction microdisk modulator is used to modulate the signal. A silicon tunable microring filter is designed to pick up the modulated signal, and a Ge photodetector is used to capture the transmitted signal. The functionality of the datalink is demonstrated by data transmission as a proof-of-concept at kHz speed level. Modulation and signal transmission results are shown with potential for high-speed operation of more than 1 Gbps. These results pave the way for monolithic integration of amplifiers and lasers, potentially for free-space communication and many other applications on a full silicon photonic platform.

## Methods

### Fabrication process

**Figure 6:**
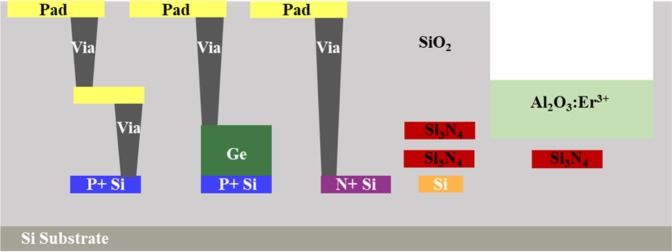
Simplified diagram illustrating the silicon photonic platform utilized for the link system. The platform includes two Si_3_N_4_ layers, a silicon layer with different doping levels, two metal layers, two via layers, a Ge layer for the photodetector, and a trench for deposition of the erbium-doped Al_2_O_3_ thin film.

The wafer-level fabrication process (except the Al_2_O_3_:Er^3+^ thin film deposition) is done in a state-of-the-art CMOS foundry at the Colleges of Nanoscale Science and Engineering, SUNY Polytechnic Institute in Albany, NY. The platform consists of two Si_3_N_4_ layers, a silicon layer with different doping levels, two metal layers, two via layers, a Ge layer for the photodetector, and a trench for deposition of an erbium-doped Al_2_O_3_ thin film. Figure 6 shows a simplified schematic of the layers in this silicon photonic platform. The top copper metal layer is for routing and contact pads. A copper via is used between the top and bottom metal layers. The bottom copper metal layer is for routing. A bottom via is for contact to the bottom silicon layer. The CMOS foundry uses 193 nm immersion lithography on a 300-mm-diameter SOI wafer with 220 nm silicon height and 2-µm thick buried oxide. The Ge layer is hetero-epitaxially grown on top of a heavily p-doped silicon base. An n-type dopant is implanted at the top of the Ge to form a vertical p-i-n junction for the detector. The vias are used to connect the n-doped Ge and p-doped silicon together with metal contact pads. Two 200-nm-thick Si_3_N_4_ layers are deposited using a plasma-enhanced chemical vapor deposition process, polished using a top surface polishing process to reduce optical scattering loss, and patterned using 193 nm immersion lithography (this bottom-up process distinguishes from the top-down etching processes^68^). The bottom Si_3_N_4_ layer defines the grating and DBR cavity for the erbium-doped laser. A 4-µm-thick silicon dioxide layer is deposited above the top Si_3_N_4_, and a 4-µm-deep trench for deposition of the gain media is etched into the silicon dioxide using the top nitride layer as an etch stop.

After the wafer-level fabrication, the wafer is diced and the back-end-of-line deposition of the gain film is performed on the chip level. A nominally 1100-nm-thick erbium-doped Al_2_O_3_ film is deposited on top of the chip via reactive co-sputtering, at an on-chip temperature of 310 °C. The film thickness and doping energy are optimized to ensure efficient lasing. The Er^3+^ doping concentration level is estimated to be 1.5 × 10^20^ cm^−3^. Under lower doping level the lasing power will decrease due to the reduced gain, while under higher doping level the lasing power will also decrease due to the clustering of the doping ion^69,70^. More details about the Al_2_O_3_ thin film deposition are provided in Supporting Information section 1.

### Numerical simulation

Effective indices and guided modes are simulated using a vector finite-difference 2D eigenmode solver, with a discretization of 20 nm. The code is written in Matlab, and it solves the wave equation of the transverse electric field at the signal wavelength. The refractive indices for Al_2_O_3_, Si_3_N_4_, and SiO_2_ are 1.649, 1.950, and 1.444, respectively. More detail on the simulation is provided in Supporting Information section 2 and the mode solver code can be found in the appendix of^71^.

## Supplementary information

Supporting Information.
