# Supplementary material for: A Silicon Photonic Data Link with a Monolithic Erbium-Doped Laser
Source: Sci Rep. 2020 Jan 24;10:1114. doi: 10.1038/s41598-020-57928-5 (PMC6981124; doi:10.1038/s41598-020-57928-5)
Supplement: Supplementary file 1 — Supporting Information. [file 41598_2020_57928_MOESM1_ESM.pdf]

Supporting Information

A Silicon Photonic Data Link with a Monolithic Erbium-Doped Laser

Nanxi Li[1,2,3], Ming Xin[1], Zhan Su[1,4], Emir Salih Magden[1,5], Neetesh Singh[1], Jelena Notaros[1], Erman Timurdogan[1,4], Purnawirman Purnawirman[1], Jonathan D. B. Bradley[1,6], and Michael R. Watts[1,*]

[1]Research Laboratory of Electronics, Massachusetts Institute of Technology, 77 Massachusetts Avenue, Cambridge, MA 02139, USA
[2]John A. Paulson School of Engineering and Applied Science, Harvard University, 29 Oxford Street, Cambridge, MA 02138, USA
[3]Current address: Institute of Microelectronics, Agency for Science, Technology and Research (A*STAR), 2 Fusionopolis Way, Singapore, 138634, Singapore
[4]Current address: Analog Photonics, 1 Marina Park Drive, Boston, MA 02210, USA
[5]Current address: Department of Electrical and Electronics Engineering, Koç University, Sarıyer, İstanbul 34450, Turkey
[6]Current address: Department of Engineering Physics, McMaster University, 1280 Main Street West, Hamilton, Ontario L8S 4L7, Canada
*Corresponding author: mwatts@mit.edu

1. Al_2O_3 Deposition Process for Integrated Laser

The integrated laser devices developed within our research group are based on rare-earth-doped Al_2O_3 thin film deposited at the top of wafers fabricated by a CMOS foundry. Thus far, laser devices based on this platform have been demonstrated by using different dopants at various wavelengths, including ytterbium at 1.0 µm, erbium at 1.5 µm, thulium at 1.9 µm, and holmium at 2.1 µm. The gain film for laser is deposited at MIT Microsystems Technology Laboratory. Figure S1 shows the deposition machine and the schematic of the deposition chamber.

For each deposition run, we include a witness sample, which has 6 µm thermal silicon dioxide at the top of silicon wafer. In order to measure the film thickness, refractive index and loss of the film deposited, the witness sample is later placed on Metricon system for characterization after deposition. The laser signal at 633 nm is used. The refractive index of the film at 633 nm is typically 1.66 to 1.67. A good passive film, or erbium doped active film should have no loss at 633 nm (or low loss below the measurement range of the Metricon system). More details about the loss and gain of the erbium doped active film are provided in the literature [1,2].

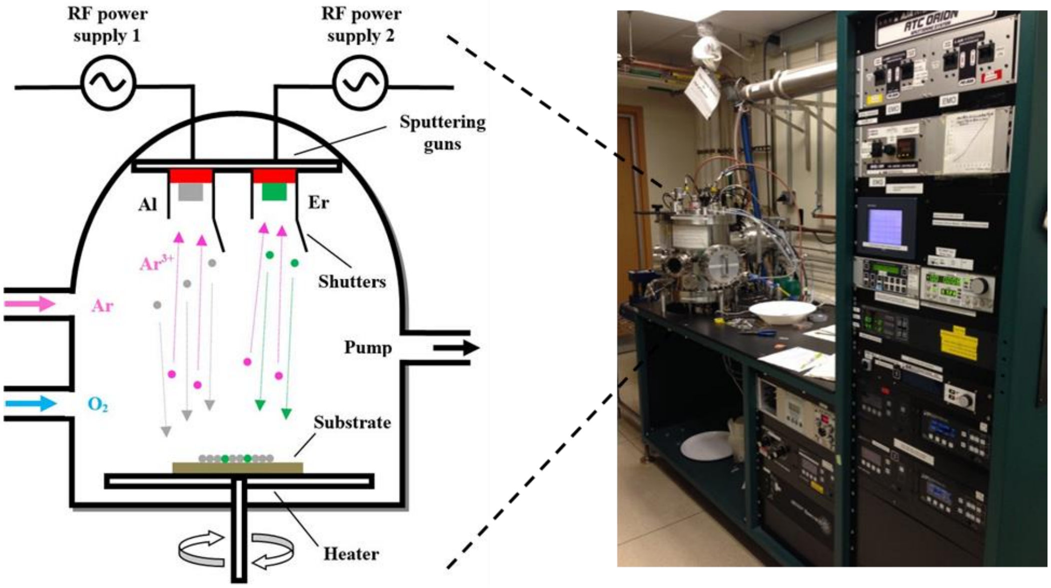

Figure S1. Photo of deposition machine with enlarged schematic of deposition chamber.

On the AJA sputter (ATC Orion) deposition machine, there is a temperature controller to set the temperature for deposition. An internal thermal couple at the bottom of the stage is in touch with a transparent quartz to monitor the temperature of the quartz. At the same time, it gives feedback to the heater lamps to stabilize it to the setting temperature. From deposition, it is found that there is an offset between the setting temperature and the temperature on the chip substrate. In order to measure the chip substrate temperature, an external thermal couple is kept in touch with the chip substrate, as shown in Fig. S2(a). High temperature silver thermal paste is applied to ensure good thermal conductivity between the external thermal couple and chip substrate. The temperature calibration, as shown in Fig. S2(b), provides the measured temperature on chip substrate vs. the setting temperature on the temperature controller. It has been found that for deposition on wafer with metal heater, the setting temperature can be 535 ^{o}C, which corresponds to 397 ^{o}C on the wafer substrate. For deposition on wafer with doped Si heater, the setting temperature can be as low as 465 ^{o}C, which corresponds to 310 ^{o}C on the wafer substrate. These setting temperatures are determined by successive deposition iterations from high to low temperature, until there is no change on the contact resistance or no significant degradation to the I-V characteristics of the active components including modulator and photodetector before and after the deposition. Finally, in order to keep the consistency of the deposition temperature on chip, quarterly temperature calibration can be done using the setup shown in Fig. S2(a).

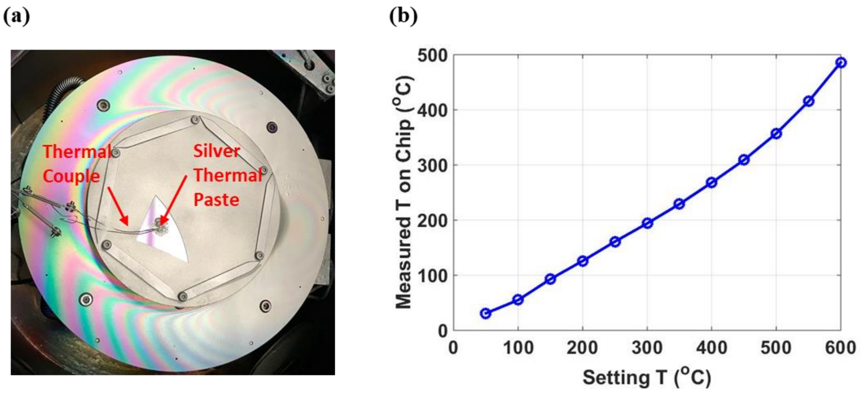

Figure S2. (a) Chip substrate temperature measurement setup: an external thermal couple is in thermal contact with chip substrate through silver thermal paste. (b) Measured temperature on chip substrate using external thermal couple vs. the setting temperature of deposition machine.

For the chips with the integrated heater, the metal pads need to be kept open after the deposition. Hence, there is a need to have area selective deposition on the chip. The solution here is to use thin glass plate to coverup the area that does not need the Al_2O_3 thin film. Two partially covered chips are shown in Fig. S3 below. The metal pads are placed on one side of the chip in the mask layout. There is one more chip on the right side of Fig. S3 to hold the thin glass plate. Two thick glass plates are placed at the top of the thin glass plate just to ensure the mechanical stability. This is an effective and simple way to achieve area selective deposition physically. Our group also tried to achieve the area selection chemically by doing the deposition on the whole chip first, then partially etch away the Al_2O_3 thin film to make the pads open. The problem with the chemical method is the residual photoresist within the trench causes the laser to non-working status. Therefore, the physical method is recommended instead of the chemical method. In the case that the metal pads are located all over the chip instead of in the same area on the chip, a thin metallic mask can be used to cover the chip with additional alignment to achieve area selective deposition.

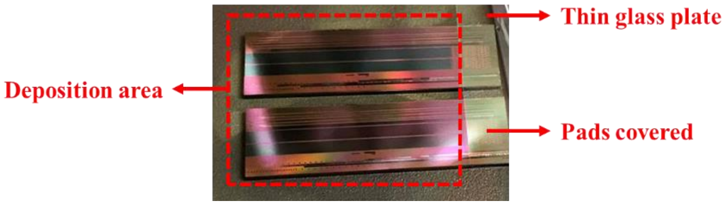

Figure S3. Two chips (on the left side) within the deposition chamber with metal pads area covered by thin glass plate.

2. Numerical simulation

The laser grating numerical simulation methodology is summarized here. The effective indices and guided modes in waveguides are simulated by vector finite-difference 2D eigenmode solver, with a discretization of 20 nm. The code is written in Matlab, and it solves the following wave equation of the transverse electric (TE) field:

$$\nabla^2 \overline{e_t} + \nabla\left(\frac{1}{n^2}\nabla(n^2)\overline{e_t}\right) + n^2k^2\overline{e_t} = \beta^2\overline{e_t}$$

where k is the wave number, n is the refractive index. After solving the above eigen problem, the square of propagation constant β^2 can be obtained from the eigenvalue, and the effective index can be calculated using $n_{eff} = \beta\lambda_0/2\pi$. The grating period is then calculated by substituting the n_{eff} obtained into the following equation:

$$\Lambda = \frac{\lambda}{2n_{eff}}$$

where λ is the designed lasing wavelength. The grating strength or coupling coefficient (κ) is calculated using the following equation [3].

$$\kappa = \frac{k^2}{2\pi\beta}(n_{SiN}^2 - n_{SiO}^2)\sin(\pi D)\tau$$

where D is grating duty cycle, which equals to 0.5, and τ is the mode overlap within the grating region.

3. Spectrum response and high-speed measurement setup

The drawings of the characterization setups for both passive spectrum response and high-speed measurement are shown in Fig. S4 (a) and (b) respectively. The model numbers of the key components are provided in the figure caption.

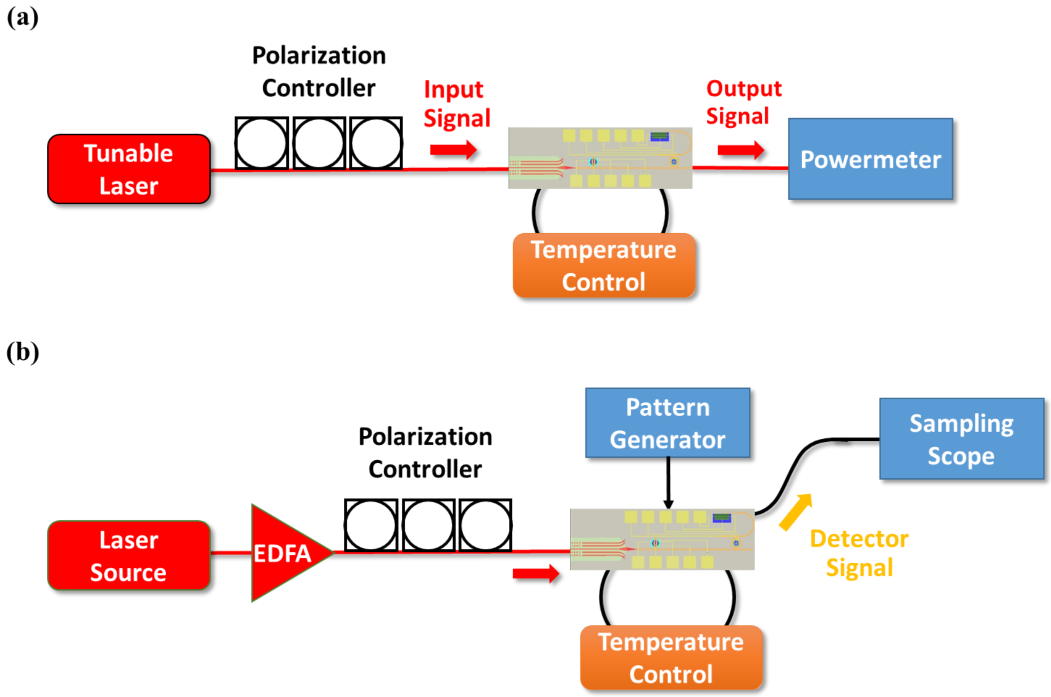

Figure S4. (a) External laser measurement setup, including a tunable laser (Keysight 81600B) to sweep the wavelength of the input signal, a polarization controller to ensure the input is coupled into the TE mode of the DBR laser and Si waveguide, an optical power meter (Keysight B1635A) to record the signals from the through port of the detector microring filter, and a TEC temperature control to modify, monitor, and stabilize the operating temperature of the system. (b) High-speed measurement setup, including a laser source cascaded with a high power EDFA (Optilab EYDFA-L-40-1), a pattern generator (Pattern Pro 12072) to provide a PRBS signal and a sampling scope (Agilent 86100D) to capture the eye diagram of the system. For this experiment, an undoped Al_2O_3 thin film rather than an Al_2O_3:Er^{3+} active film is deposited on the chip with the same design to form the passive laser waveguide without signal absorption from the erbium ions.

4. Full link loss budget

In order to have a better understanding of the optical loss in the link system. We conducted the following loss budget analysis. The on-chip optical power at the lasing wavelength from the 980 nm pump input side of the erbium laser is estimated to be -26 dBm, with all the coupling losses calibrated out. For the DBR laser, the grating transmission at the output side is designed to be 2 times higher than the input side. Hence, the on-chip optical power at the lasing wavelength from the output side can be estimated to be -23 dBm, pumped by 60 mW 980 nm pump power launched on-chip. The estimated laser slope efficiency is 0.02%. The photocurrent measured at the Ge detector is around 20 nA. For our typical Ge photodetector, the responsivity is calibrated to be 1A/W. 20 nA corresponds to 20 nW of optical power, which is -47 dBm. Hence, the total optical coupling loss from the erbium-doped DBR laser output to the Ge photodetector is around 24 dB. This total loss includes the adiabatic transitions from the laser gain waveguide to a single Si_3N_4 waveguide, from the single Si_3N_4 waveguide down to the Si waveguide, the loss in Si modulator, Si microring filter, and the taper to the Ge detector. As the Si modulator, the

microring filter, and the Ge photodetector are taken from existing components with minor variations [4-7], most of the loss should be contributed by the coupler from laser to the waveguide. For future designs, such 24 dB total loss is expected to be reduced to below 10 dB by optimizing the design for the waveguide transitions and the wafer-level waveguide fabrication process. In addition, the wall-plug efficiencies of stand-alone III-V semiconductor lasers working in the communication band around 1550 nm are reported to be up to 4.2% [8-10]. The integration of these lasers is achieved through chip-scale bonding processes. In comparison, our laser integration is a single-step physical vapor deposition process, which is scalable to the wafer level and compatible with CMOS fabrication. Although the current DBR laser efficiency is low, it can be improved. The low efficiency is mainly attributed to the reduced gain film deposition temperature and the roughness at the bottom of the gain trench due to the voids in the nitride cladding oxide. In the near future, the erbium-doped gain film deposition and the trench fabrication process can be optimized to enhance the laser output power and, hence, improve the laser slope efficiency (for example, a substrate bias voltage could be added during the gain film deposition process to compensate for the reduced deposition temperature).

5. References

1. Purnawirman, Ph.D. Thesis, Department of Electrical Engineering and Computer Science, Massachusetts Institute of Technology, Cambridge (2017).
2. K. Shtyrkova, Ph.D. Thesis, Department of Electrical Engineering and Computer Science, Massachusetts Institute of Technology, Cambridge (2018).
3. T. E. Murphy, Ph.D. Thesis, Department of Electrical Engineering and Computer Science, Massachusetts Institute of Technology, Cambridge (2001).
4. Li, N. *et al.* C-band swept wavelength erbium-doped fiber laser with a high-Q tunableinterior-ridge silicon microring cavity. *Opt. Express* **24**, 22741-22748 (2016).
5. Timurdogan, E. *et al*. A High-Q Tunable Interior-Ridge Microring Filter. *Conference on Lasers and Electro-Optics (CLEO),* San Jose, California, 2014. p. SF2O.3.
6. Timurdogan, E. *et al.* An Ultra Low Power 3D Integrated Intra-Chip Silicon Electronic-Photonic Link. *Optical Fiber Communication Conference 2015 Post Deadline Papers.* p. Th5B.8.
7. Byrd, M. J. *et al*. Mode-evolution-based coupler for high saturation power Ge-on-Si photodetectors. *Opt. Lett.* **42**, 851–854 (2017).
8. S. Lin, *et al.* Efficient, tunable flip-chip-integrated III-V/Si hybrid external-cavity laser array. *Opt. Express*, **24**, 19, 21454-21462 (2016).
9. B. Song, *et al.* High-Thermal Performance 3D Hybrid Silicon Lasers. *IEEE Photon. Technol. Lett.*, **29**, 14, 1143, (2017).
10. H. Guan, *et al.* Widely-tunable, narrow-linewidth III-V/silicon hybrid external-cavity laser for coherent communication. *Opt. Express*, **26**, 7, 7920-7933 (2018).
